# Disentangling the Role of Climate, Topography and Vegetation in Species Richness Gradients

**DOI:** 10.1371/journal.pone.0152468

**Published:** 2016-03-25

**Authors:** Mario R. Moura, Fabricio Villalobos, Gabriel C. Costa, Paulo C. A. Garcia

**Affiliations:** 1 Departamento de Zoologia, Instituto de Ciências Biológicas, Universidade Federal de Minas Gerais, Belo Horizonte, Minas Gerais, Brazil; 2 Red de Biologia Evolutiva, Instituto de Ecologia A.C., Veracruz, Mexico; 3 Departamento de Ecologia, Centro de Biociências, Universidade Federal do Rio Grande do Norte, Natal, Rio Grande do Norte, Brazil; University of Sydney, AUSTRALIA

## Abstract

Environmental gradients (EG) related to climate, topography and vegetation are among the most important drivers of broad scale patterns of species richness. However, these different EG do not necessarily drive species richness in similar ways, potentially presenting synergistic associations when driving species richness. Understanding the synergism among EG allows us to address key questions arising from the effects of global climate and land use changes on biodiversity. Herein, we use variation partitioning (also know as commonality analysis) to disentangle unique and shared contributions of different EG in explaining species richness of Neotropical vertebrates. We use three broad sets of predictors to represent the environmental variability in (i) climate (annual mean temperature, temperature annual range, annual precipitation and precipitation range), (ii) topography (mean elevation, range and coefficient of variation of elevation), and (iii) vegetation (land cover diversity, standard deviation and range of forest canopy height). The shared contribution between two types of EG is used to quantify synergistic processes operating among EG, offering new perspectives on the causal relationships driving species richness. To account for spatially structured processes, we use Spatial EigenVector Mapping models. We perform analyses across groups with distinct dispersal abilities (amphibians, non-volant mammals, bats and birds) and discuss the influence of vagility on the partitioning results. Our findings indicate that broad scale patterns of vertebrate richness are mainly affected by the synergism between climate and vegetation, followed by the unique contribution of climate. Climatic factors were relatively more important in explaining species richness of good dispersers. Most of the variation in vegetation that explains vertebrate richness is climatically structured, supporting the productivity hypothesis. Further, the weak synergism between topography and vegetation urges caution when using topographic complexity as a surrogate of habitat (vegetation) heterogeneity.

## Introduction

Biodiversity gradients are the result of ecological and evolutionary processes acting at multiple spatial and temporal scales [1]. On the one hand, evolutionary processes such as speciation, extinction, and biogeographic dispersal, contribute to shape biodiversity patterns, adding or removing species across time [2]. On the other hand, ecological processes are considered over contemporary time scales and include current climate, productivity, and environmental heterogeneity, among others [3]. Most of these ecological processes are associated to environmental gradients (EG), which can be separated into biotic or abiotic factors. It is important to highlight that the term ‘biotic’ here is not used in the sense of biotic interactions such as competition and predation. Instead, biotic factors refer to gradients related to land cover and vegetation structure, whereas abiotic factors refers to climatic and topographic gradients [4]. In general, studies trying to explain species richness only use abiotic factors [5–7], and rarely consider the effects of biotic factors [8, 9]. However, the inclusion of biotic factors in macroecological studies has attracted attention from ecologists. For example, species richness of butterflies, amphibians, reptiles and birds, at a 100-km^2^ mosaic in Madrid, were more influenced by biotic factors (measured as the number of land use classes and proportion of specific habitats) than by elevation [8]. Similarly, butterfly richness in Canada was best predicted by the number of land cover classes, with a smaller but complementary role of climatic and topographic factors [9]. Moreover, bird richness in North America was more correlated with vegetation properties than climate and topography [10].

**Fig 1:**
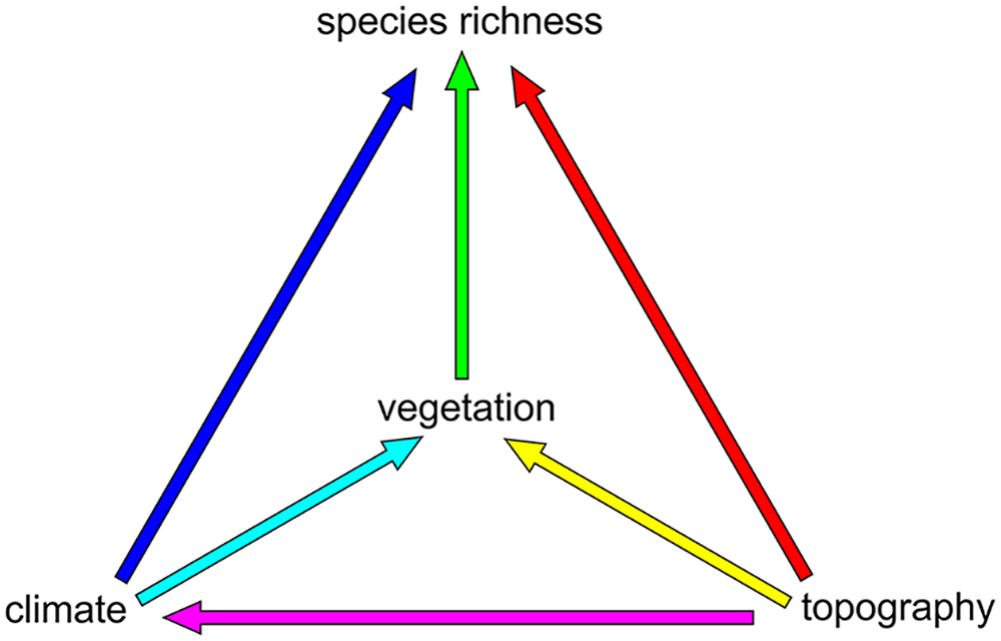
The synergism between environmental gradients (EG) driving biodiversity patterns. Arrows indicate causal assumptions among EG and species richness. Topographic gradients act on diversity components via indirect links with climate and vegetation, while climatic gradients act indirectly through their effect on vegetation.

Climatic, topographic and biotic gradients are naturally related to each other. Climate can affect species richness indirectly via their effects on vegetation [4], while topography can interplay with both climate and vegetation, also affecting species richness [4, 11]. Since different EG may have common effects on species richness, it is interesting to assess their unique and shared contributions when trying to explain biodiversity patterns [12]. These climatic, topographic and biotic gradients do not necessarily drive species richness in similar ways [13] and therefore uncovering their relative importance is fundamental to improving our understanding of the effect of global climate and land use changes on biodiversity patterns [14, 15]. Many of the ecological hypotheses traditionally invoked to explain species richness patterns indirectly rely on synergistic associations among EG. For instance, the‘productivity hypothesis’ states that the energy input captured by plants is converted in food resources, and the biomass available through trophic cascades ultimately affect animal richness [16]. Following this hypothesis, one may expect to observe the synergistic association between climate and vegetation in explaining species richness. The ambient-energy hypothesis is based on the assumption that physiological requirements determine an organisms distribution. Thermoregulation constraints can be imposed solely by current climate or through the synergistic association between climate and topography [11, 17]. In addition, different synergistic associations may be expected under the habitat heterogeneity hypothesis. For example, the synergism between topography and vegetation may increase resource diversity and structural complexity. Such association could promote species coexistence and persistence, allowing more diverse communities to develop [4, 18]. Therefore, the influence of climatic, topographic and biotic factors on species richness patterns can be linked through hypothetical causal relationships (Fig 1).

The role of EG to explain species richness can also differ according to inherent characteristics of the organisms under study. Dispersal ability, for example, is intrinsically related to the organism’s capability to explore the environmental heterogeneity of its surroundings [19]. Species with greater dispersal ability can more promptly adjust their geographical distribution in response to climate change [20], potentially resulting in equilibrium between the distribution of good dispersers and current climate conditions [21]. Likewise, highly vagile species could have a better perception of landscape features (*i.e*. biotic factors) and thus modify their distribution due to land use changes accordingly [18, 22]. In contrast, less vagile species would be more sensitive to topographical features that can impose barriers to dispersal [23]. Therefore, the role of climatic, topographic and biotic factors driving species richness patterns of taxa with distinct dispersal abilities may not be the same. In this paper, we determine the synergistic associations between climatic, topographic and biotic gradients, and use our results to evaluate three non-mutually exclusive hypotheses (productivity, ambient-energy and habitat heterogeneity) often proposed to explain broad scale patterns of species richness. We predict that species richness of the most vagile groups would be better explained by climatic factors, followed by biotic ones. Conversely, topographic factors should be relatively more important to explain species richness of poor disperses. To shed light on this question, we compare our findings across vertebrate groups with distinct dispersal abilities to discern whether organisms’ vagility affects the relative influence of such gradients when explaining species richness.

## Methods

### Study area

The geographical extent of this study is the Neotropical region, excluding all islands with the exception of Caribbean Islands. The Neotropics are recognized for their high biodiversity and harbor nearly a third of the world’s biodiversity hotspots [24]. We mapped the Neotropical region using an equal area projection and overlaid a grid cell of 110 × 110 km (ca. 1° × 1° at the equator) of spatial resolution. We excluded the coastal cells with <50% of terrestrial cover, adding up to 1679 remaining cells (regional cells, hereafter).

### Species data

Volant animals are better dispersers than terrestrial animals [25], and endotherms are better dispersers than ectotherms [26]. Therefore, to consider groups of low, intermediate and high dispersal abilities we used distributional data on amphibians, mammals and birds, respectively. Species distributional data were obtained from digital databases of amphibians, mammals and birds, available at BirdLife International (http://www.birdlife.org/) and International Union for Conservation of Nature’s (http://www.iucnredlist.org) portals. Often these maps apply minimum convex polygons around interpolated species presence records and may include false-presences and commission errors [27], making them usable at grains of ca. 100 km and coarser [28]. Nonetheless, these databases currently represent the most comprehensive maps of vertebrate geographical distribution within the Neotropical realm, allowing primary investigations until fine-resolution distribution data become available [29, 30].

Previous studies have shown that volant and non-volant mammals differ in their patterns of species richness and responses to ecological gradients [31]. Since we aimed to detect potential differences in relative importance of environmental constraints related to dispersal ability, we performed analyses separately for volant and non-volant terrestrial mammals. We excluded marine mammals from our analyses. For simplicity, we refer to these four groups as ‘vertebrates’ throughout the text. To determine species richness of amphibians, terrestrial mammals (non-volant and volant), and birds in the Neotropics, we rasterized each species range polygon at 110 × 110 km spatial resolution. We included any species with any part of its distribution in the terrestrial portion of the Neotropical realm, resulting in 3043 species of amphibians, 1540 mammals (1218 non-volant and 322 volant) and 4041 birds. All calculations were performed in R 3.1.2 [32].

### Measuring biotic factors

**Fig 2:**
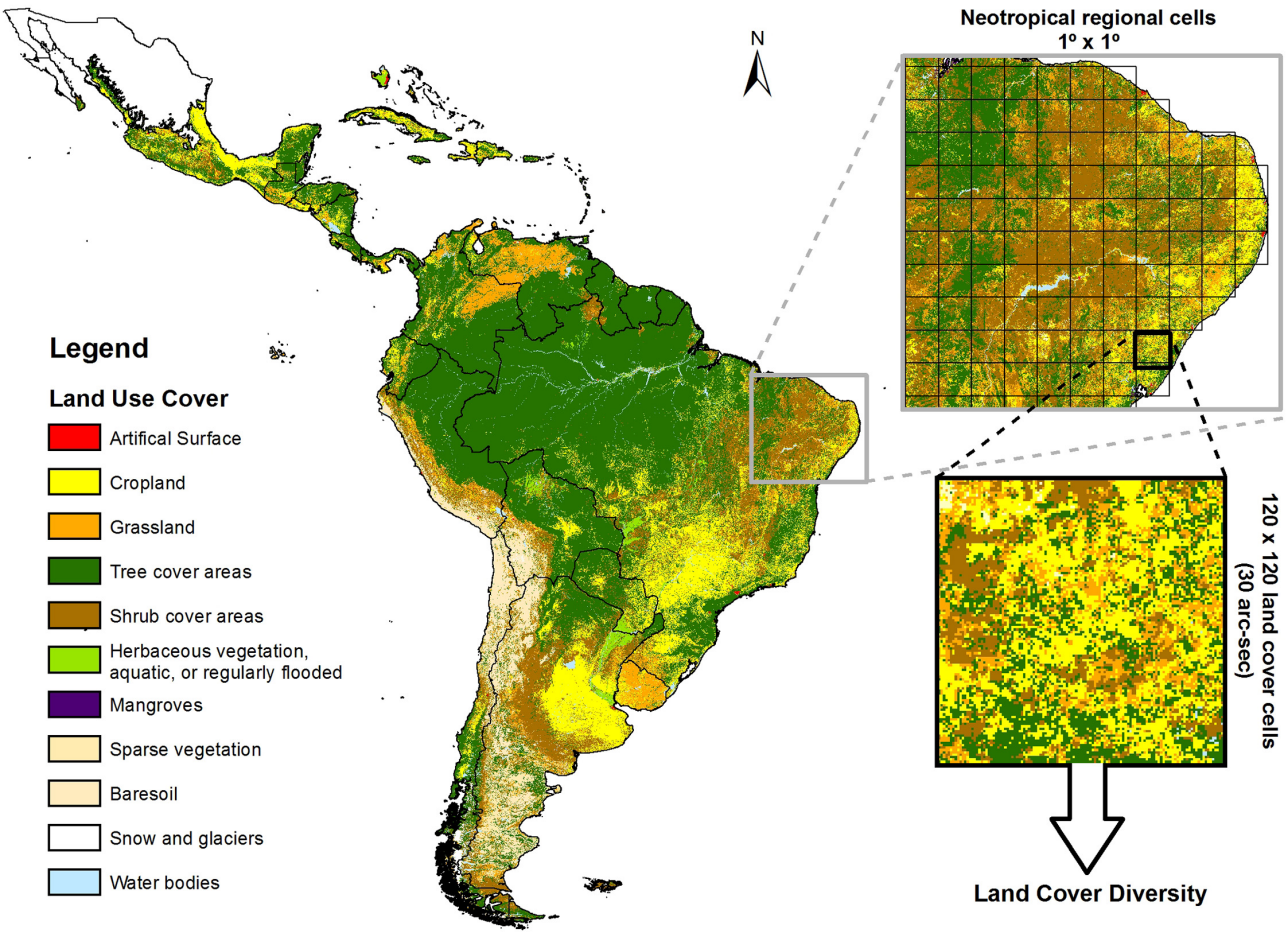
Land use map for Neotropical realm obtained through the GLC-SHARE database. For each 1° × 1° grid cell, the land cover diversity was extracted as the Shannon index of land cover classes.

Biotic factors are usually represented by measures of heterogeneity in landscape composition, such as diversity indexes (e.g. Shannon, Simpson) calculated from proportion of land use classes within a given region [22]. Here, we used the *Global Land Cover-SHARE* database (GLC-SHARE, [33]) to obtain a measure of land cover diversity. The GLC-SHARE database provides the percentage coverage for 11 land use categories at 30 arc-sec (≈1 km^2^) resolution. For each pixel, values from 0–100% represent the coverage of each land use category. Using the Highest Position tool on ArcGIS 9.3, we grouped the 11 GLC-SHARE layers to build a single layer, which incorporated the predominant land cover (i.e. the layer with the highest percentage value) in each 30 arc-sec pixel. We used the Tabulate Area tool to count pixels of each land use category in this layer within the regional cells. Then, we measured the land cover diversity as the Shannon index of GLC classes (Fig 2). Computations were performed in R 3.1.2 [32], using the *vegan* package [34].

**Fig 3:**
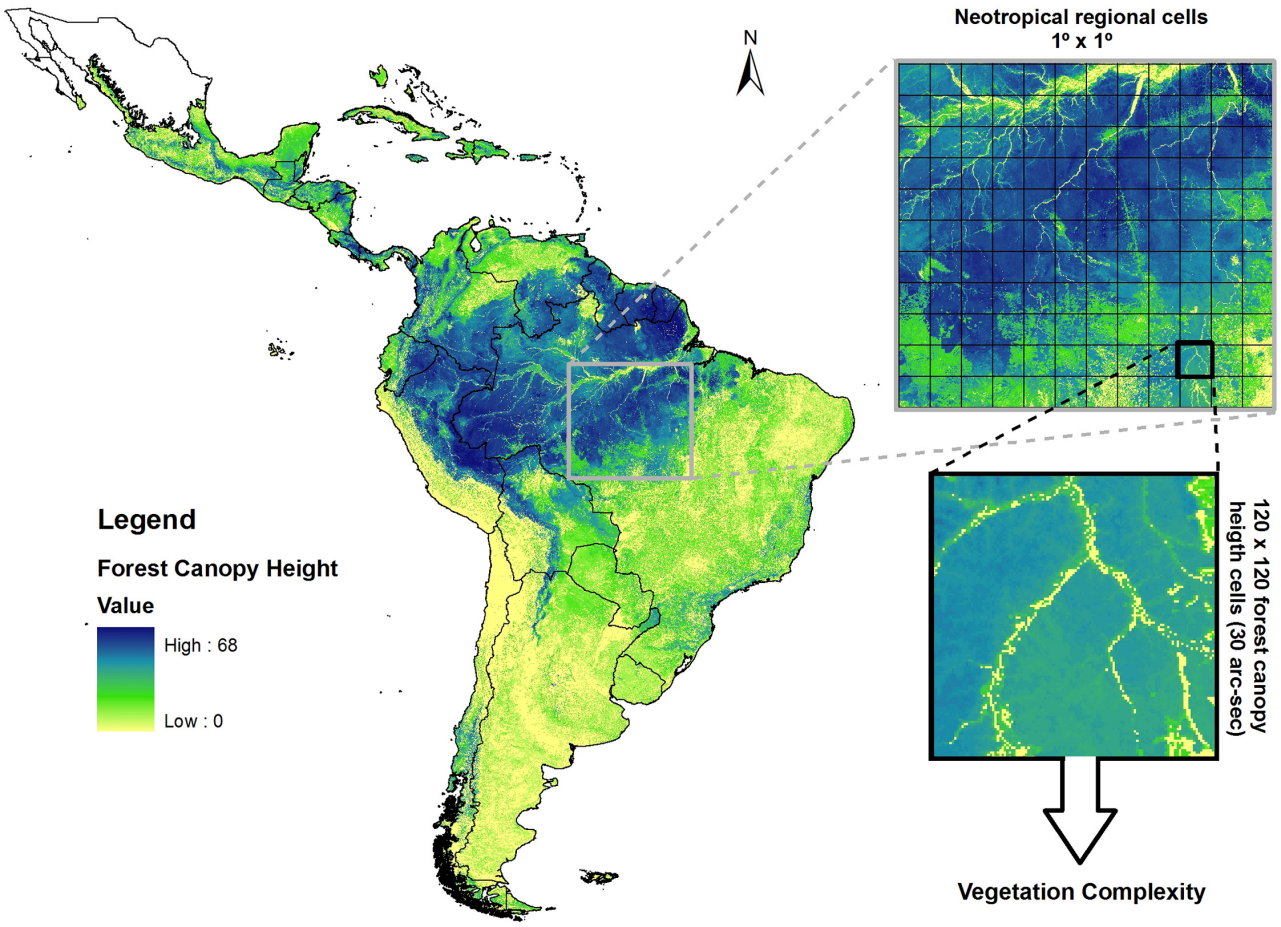
Forest canopy height for Neotropical region obtained through the 3D Global Vegetation Map database. For each 1° × 1° grid cell, two measures of vegetation complexity were extracted: (i) standard deviation of forest canopy height and (ii) forest canopy height range.

Another important biotic factor is vegetation complexity, usually characterized as the richness or diversity of plants, plant density, or vegetation height [4]. We used *The 3D Global Vegetation Map* database [35] to acquire a measure of vegetation complexity. This database represents a global model for forest canopy height at 30 arc-sec resolution. We used the Zonal Statistic tool from ArcGIS 9.3 to obtain two vegetation complexity measures: (i) standard deviation of forest canopy height and (ii) forest canopy height range (Fig 3).

### Measuring abiotic factors

To account for climatic factors we used four variables: (i) annual mean temperature, (ii) annual precipitation, (iii) temperature annual range (= max temperature of warmest month—min temperature of coldest month) and (iv) precipitation range, represented as the difference between precipitation of the wettest quarter and precipitation of the driest quarter. All climatic variables were downloaded from Worldclim database [36] at 30 arc-sec resolution. For each variable, we calculated the average value for each regional cell using the Zonal Statistics tool on ArcGIS 9.3.

We used the SRTM database [37] at 30 arc-sec resolution and the Zonal Statistics tool on ArcGIS 9.3 to obtain three measures of topographic factors: (i) mean elevation, (ii) elevational range, and (iii) coefficient of variation of elevation (roughness).

### Data analysis

To verify the relative importance of distinct EG, we initially separated the explanatory variables into three distinct predictor sets according to (i) topographic, (ii) climatic, or (iii) biotic factors. To account for hump-shaped relationships, we included linear and quadratic terms of each EG in the respective predictor set. The use of all environmental variables inevitably increases the multicollinearity. Although multicollinearity is not a problem to model prediction, it inflates the standard error of model parameters, leading to unreliable and unstable estimates of regression coefficients [38]. That is, small changes in the data may result in large changes in the model coefficients, and the extrapolation of results beyond our study area is prone to errors [39]. We minimized multicollinearity by performing a Principal Component Analysis (PCA) separately in each predictor set, and extracting the three first axes of each PCA to use as environmental variables. These PCA axes accounted for 91.6% of the variation in the climatic set, 97.0% topographic set, and 98.8% biotic set (S1 Table), also presenting low multicollinearity (VIF < 2.6 for all PCA-based variables, S2 Table.

A common approach to disentangle the contribution of distinct factors is variation partitioning (also known as commonality analysis [40]). This technique allows the assessment of the unique and shared contributions of different predictors (or sets of predictors) in explaining a particular response variable [38]. The shared contribution between two predictors (or sets of predictors) can then be used to identify synergistic processes operating between these predictors (or sets of predictors) [41]. At this point, we can adopt a simplistic but useful interpretation regarding how species richness can be directly or indirectly affected by different EG. It is reasonable to assume that shared contributions between two types of gradients obtained via variation partitioning may represent the synergistic association between them, and therefore an indirect link supporting the causal relationships between these gradients and species richness. Therefore, we used variation partitioning based on ordinary least squares (OLS) models [42] to obtain the relative importance of each predictor set to explain variation in species richness.

The presence of spatial autocorrelation in an OLS model residuals violates the independence assumption and biases estimation of standard errors coefficients [38]. We examined the spatial structure in our OLS model residuals through spatial correlograms of Moran’s *I* coefficients, calculated at 21 geographic distance classes [38, 43]. As substantial spatial autocorrelation was detected, we incorporated the spatial structure into the OLS models by applying an eigenvector spatial filtering analysis (also known as Spatial EigenVector Mapping—SEVM)[44] on the OLS residuals of each model built. This technique is based on the eigenfunction decomposition of a spatial geographical distance matrix [44, 45]. The eigenvectors extracted from this matrix were used as explanatory variables (spatial filters incorporated into the respective OLS model) to reduce spatial trends in the OLS residuals [46]. Although there are several ways to generate connectivity matrices for SEVM, we follow recommendations in [47], based on the maximum distance that keeps all sites linked, which is produced on the basis of a minimum spanning tree (≈450 km for all models). For each OLS model, we selected the spatial filters to minimize the Moran’s *I* below the 0.1 threshold and then used as the spatial set of variables in the variation partitioning analysis. Spatial filters were generated and applied separately for the species richness of each vertebrate group (see S1 Fig for correlograms).

For each vertebrate group (amphibians, non-volant mammals, bats and birds), we obtained the percentage of variance in species richness uniquely explained by each set of variables, as well as the shared explained variation among these sets, and the percentage of the variation unexplained. Note that by unique contribution/fraction of a particular environmental set (biotic, climatic, or topographic), we are referring to the fraction of variation in species richness that is explained by the respective environmental set but is not structured within any other set. By unique spatial fraction we are referring to the variation in species richness that is explained by the spatial set and is not shared with the environmental sets. By shared fraction, we are referring to the amount of variation that two or more predictor sets have in common with the species richness (i.e. dependent variable) and it should not be confused with interaction effects (e.g. in GLM, ANOVA). By the ‘total importance’ of a particular predictor set, we are referring to the sum of all fractions related to that predictor set, including those fractions shared with the other predictor sets (see [38] for details on the interpretation of variation partitioning fractions).

We included cells with zero values of richness, but results were similar when they were excluded. All species richness measures were log(*x* + 1) transformed before analyses. Moran’s *I* correlograms, OLS and SEVM analyses were performed in SAM v4.0 [48]. Principal component analysis and variation partitioning were performed in R 3.1.2 [32] using the *vegan* package [34].

## Results

**Fig 4:**
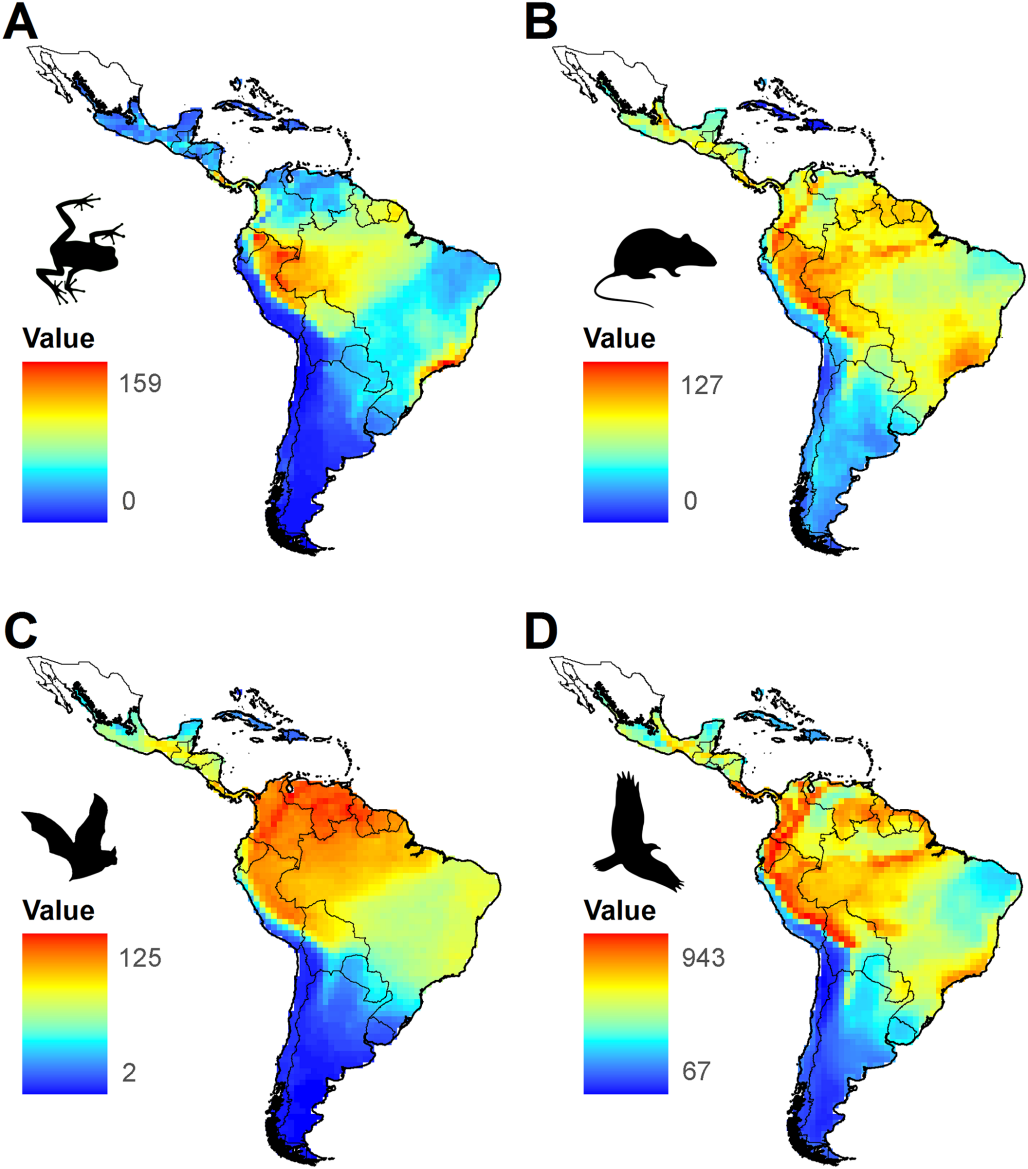
Vertebrate richness patterns in the Neotropical realm. Species richness of amphibians (A), non-volant mammals (B), bats (C), and birds (D).

The overall species richness was higher for birds (mean ± SD = 408.5 ± 163.9) than mammals (61.0 ± 23.6 non-volant and 64.0 ± 37.2 volant mammals) and amphibians (47.8 ± 33.4) (Fig 4). Bird and bat species richness were most highly correlated (*r* = 0.914), followed by bird and amphibian richness (*r* = 0.909), bats and amphibians (*r* = 0.875), non-volant mammals and birds (*r* = 0.825), non-volant mammals and amphibians (*r* = 0.805), and then non-volant mammals and bats (*r* = 0.794).

**Fig 5:**
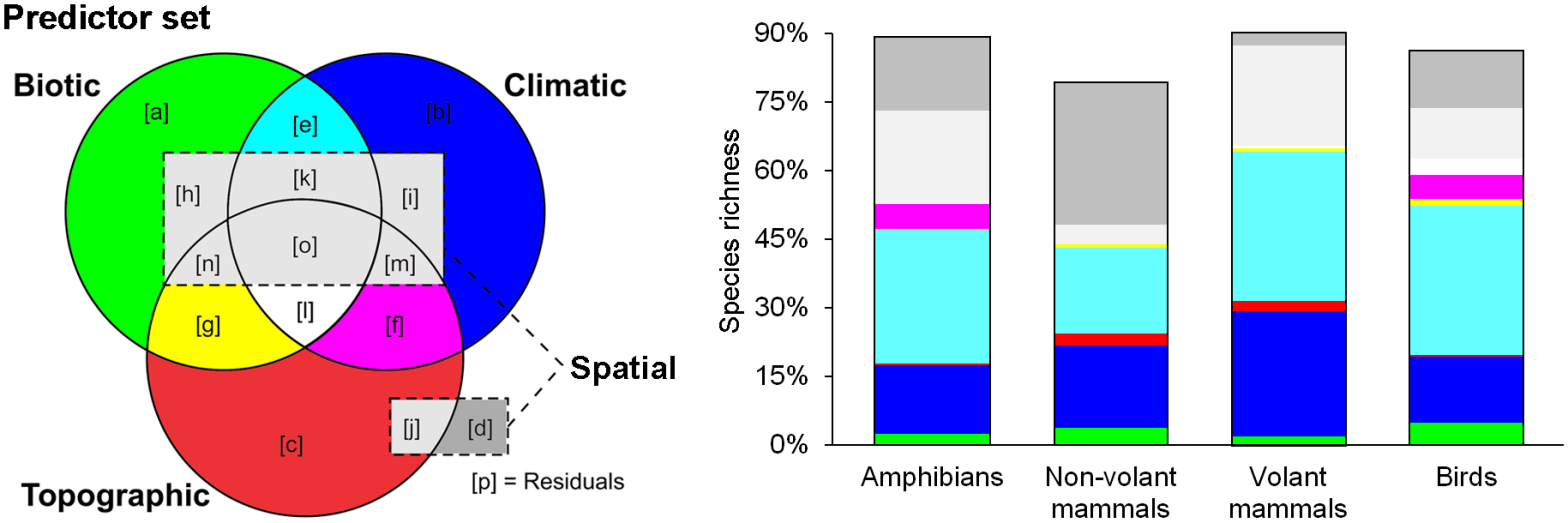
Variation in species richness explained by environmental gradients. Primary colors (red, green and blue) denote the proportion of variation explained by the unique fraction of the topographic, biotic or climatic sets. Secondary colors (yellow, cian, magenta) denote the variation commonly explained by two of the three types of environmental sets. White color indicates the variation commonly explained by biotic, climatic and topographic set. Gray colors represent the variation explained by the unique spatial fraction (dark gray) or by the shared fraction between the spatial set and any other environmental set (light gray). Unexplained variation is omitted for simplicity (see Supporting Information for further details on variation partitioning analyses). Each letter in the Venn diagram represents a fraction of the variation partitioning analysis and add up to the total set of biotic [aeghklno], climatic [befiklmo], topographic [cfgjlmno] and spatial [dhijkmno] factors.

The average variation in vertebrate species richness explained by the three environmental sets was approximately 70% (72.5%, 46.7%, 86.5% and 79.4%, respectively for amphibians, non-volant mammals, bats, and birds). The inclusion of the spatial set increased the average explained variation to 83.4%. Overall, the species richness variation explained by all four predictors sets was 88.9% for amphibians, 77.7% non-volant mammals, 92.6% bats and 86.2% birds (Fig 5). The total importance of the spatial set was higher for amphibians (36.7%) and non-volant mammals (35.4%) than for bats (28.0%) and birds (23.7%). Detailed fractions of variation partitioning are presented in S3 Table.

The total contribution of the climatic set explained on average 61.9% of vertebrate species richness. The percentage of species richness variation explained by climate decreased to nearly a third (18.7% on average) when only the unique climatic fraction was considered. Among the endotherms, the total climatic contribution was greater in good than poor dispersers (38.8% for non-volant mammals, 64.5% birds, and 78.4% bats), although it was also higher for amphibians (65.7%). The influence of dispersal ability in the relative importance of climate was less evident for the independent climatic fraction, being 15.1% for amphibians, 17.9% non-volant mammals and 27.2% volant mammals, although it was lower for birds (14.4%).

The average amount of species richness variation explained by the total topographic set was 10.9%. When only the unique fraction is considered, topography played a minor role and explained on average 1.4% of vertebrate species richness. The unique topographic contribution was lower in amphibians (0.2%) and birds (0.4%) than mammals (2.6% for non-volant and 2.4% volant mammals). However, an opposite trend was verified for the total contribution of the topographic set. The sum of all variation partitioning fractions related to topography equals 19.6% for amphibians, 14.3% for birds, and 4.4% and 5.2% for non-volant and volant mammals, respectively.

The total contribution of the biotic set explained on average 43.8% of vertebrate species richness. After accounting for the shared fractions of explained variation, the average contribution of the independent biotic fraction was 3.2%. This unique biotic fraction slightly increased from poor to good dispersers (2.4% for amphibians, 3.7% non-volant mammals, and 4.8% birds), although it was only 1.9% for bats. The relative importance of the total biotic set showed a similar trend of increasing from poor to good dispersers (31.6% for non-volant mammals, 45.4% bats, and 51.9% birds), although it was higher for amphibians (46.4%).

Among the portions of the variation in species richness that can be attributed to shared fractions of two environmental sets, the covariation between biotic and climatic factors stands out (Fig 5). The climatic-biotic fraction explained on average 28.4% of the variation in vertebrate species richness (29.4% for amphibians, 18.9% non-volant mammals, and 32.7% for both bats and birds). The contribution of the biotic-topographic fraction was extremely reduced, especially for amphibians (0.1%) and mammals (0.7%) while slightly higher for birds (1.7%). In addition, the variation in species richness explained by climatic-topographic factors together was 5.6% for amphibians, 5.2% for birds, and was negligible for mammals. Finally, the shared fraction of the variation explained by all three environmental sets was 3.5% for birds, whereas it was negligible for amphibians, non-volant and volant mammal.

## Discussion

Climatic factors best explain species richness patterns in all vertebrate groups, followed by biotic and then topographic factors. Nearly half of the explained variation attributed to climate is also shared with the biotic set. That is, most of the variation in vegetation that explains vertebrate richness is climatically structured. In general, species richness of vertebrates is similarly explained by the combined sets of environmental variables, except for non-volant mammals that shows a comparatively lower influence of EG. Although we analyzed bats and non-volant mammals separately, the results obtained for non-volant mammals may have been misinformed by the high variation in life-history traits existing among them [31]. The synergistic association between climate and vegetation has been reported for non-volant mammals in South America [31] and endotherms in other high-energy areas [49]. Such indirect effects of climate via vegetation corroborates the productivity hypothesis in explaining species richness [17], whereas the unique contribution of climate supports the ambient-energy hypothesis. The greater explanatory power of the productivity over ambient-energy hypothesis has been found in high-energy areas, whereas the opposite may be observed in low-energy areas [3].

Our findings indicate that topographic and biotic factors explain distinct fractions of the variation in species richness. The synergistic association between these two environmental sets is notably small for all vertebrate groups, even if we consider topographic and biotic sets without controlling for unknown spatially structured factors (S2 Fig, S4 Table). Because elevational gradients show a large number of correlated environmental factors that could affect plant diversity patterns [49], some studies have used topographic factors as a surrogate for habitat (biotic) heterogeneity [17, 50, 51]. However, our results indicate that the indirect link of topography to species richness via vegetation is weak or hard to detect, at the least at the scale of this study. Similar findings are reported for European mammals, suggesting that heterogeneity in habitat (land cover diversity) and topography represents distinct aspects of the environment, and therefore may affect species richness through different mechanisms [52]. Most of the support for the habitat heterogeneity hypothesis is associated to the unique contribution of the biotic set. Thus, a more cautious approach may be required when using topographic related variables as a surrogate of habitat heterogeneity.

The influence of dispersal ability in the relative importance of EG is evident among mammals. The total and unique climatic fractions better explain the species richness of volant than non-volant mammals. Indeed, it has been argued that the strong climate-richness relationship in Chiroptera is a result of high tropical niche conservatism in bats [53]. This narrower physiological tolerance coupled with high dispersal ability may explain the greater equilibrium of bat distributions to current climate [20, 21]. It is worth noting that the small contribution of climate in explaining species richness of non-volant mammals may be related to distinct evolutionary origins of mammalian clades [53]. The co-occurrence of clades adapted to tropical (*e*.*g*. Feliformia) and temperate (*e*.*g*. Caniformia, Rodentia) climates overshadow the climate-richness relationship of non-volant mammals [53, 54]. Further, we expected a resemblance in the climatic contributions for bat and bird richness, due their ability to fly. However, the smaller contribution of climate to bird richness may be related to differences in birds’ evolutionary history. The climate–richness relationship of New World birds is associated to tropical niche conservatism in basal clades, in combination with repeated broad shifts in adaptive peaks of new clades [55]. Consequently, bird species of derived and basal clades differ in responses to environmental variables [56], which may overshadow the effect of climate on the overall bird richness pattern. Also, the notably high contribution of climate in explaining amphibian richness (poor disperser) is not unexpected, since ectotherms may be particularly sensitive to climatic factors due their ecophysiology [57].

In addition, changes in elevation could be enough to impose either physical or physiological barriers to species dispersal [58, 59]. These barriers are more evident in the Tropics, where species exhibit narrow thermal tolerances due to lower seasonal variation than temperate regions [60, 61], and therefore are less able to disperse across climatic gradients due to changes in elevation than temperate species [62]. Although both mechanisms (physical and physiological) contribute to explain species richness along topographic gradients, our findings suggest the predominance of distinct mechanisms across vertebrate groups. The substantial fraction of amphibian richness explained by the shared contribution of climatic and topographic sets shows that amphibians may be more susceptible to physiological than physical limitations across topographic gradients. Alternatively, the synergism between climate and topography reduces atmospheric pressure and potentially increase wind speeds, which may restrict movements and foraging opportunities for birds [63]. In contrast, the importance of the unique topographic fraction in explaining richness of volant and non-volant mammals reflects the susceptibility of mammals to physical barriers imposed by topography. In particular, this may be the case for species with narrow to medium range sizes that have smaller body size and home range, and thus low dispersal ability [64, 65]. Our findings contrast with previous evidence for high-energy areas in North America, where mammal richness is highly correlated with topographic heterogeneity [50]. Otherwise, the small importance of topography in high-energy areas has been associated with a disproportionate contribution of wide-ranging species to overall species richness patterns [31, 66]. Since widely distributed mammals are usually good dispersers [65, 67], they may be less sensitive to topographic barriers, weakening the overall elevation-richness relationship.

Past studies have traditionally used other gradient measures to investigate hypotheses related to productivity (e.g. annual evapotranspiration–AET, net primary productivity–NPP, and normalized difference vegetation index–NDVI) [17], ambient-energy (annual potential transpiration–PET) [3], habitat heterogeneity (elevational range ‘per se’) [17, 50, 51]. Albeit these measures are appropriate under the aims of such studies, their use hinders the synergistic associations between environmental gradients. In this study, we have taken advantage of a simple tool (variation partitioning or commonality analysis) to quantify the synergistic associations between broad sets of environmental gradients. Besides disentangling the relative importance of climate, topography and vegetation, we have also deconstructed the explanatory power of productivity, ambient-energy and habitat heterogeneity hypotheses in explaining species richness. By doing so, we identify causal models that can be further explored. For instance, the synergism between topographic and vegetation might be related to the association between elevational range and land cover diversity, while the synergism between climate and vegetation could be related to forest canopy complexity and water availability [68]. The causal relationships among single predictors can be properly addressed through a Structural Equation Modeling (SEM) approach, for example.

Our findings also indicate a substantial relative importance for the spatial set (spatial filters as explanatory variables). It is worth noting that such explained variation may arise through several causes, such as: (i) environmental factors not included in our predictor sets; (ii) biotic interactions in the sense of competition and predation; (iii) spatially structured historical events, for example within ecoregions; (iv) spatial autocorrelation in our response variables, or (v) noise within our data [38]. Interestingly, the relative importance of the spatial set is higher for amphibians and non-volant mammals, suggesting a higher susceptibility of these groups to local idiosyncrasies. That is, animal groups with low to intermediary levels of dispersal ability are more affected by spatially structured processes than good dispersers, which is in line with previous evidence [69].

In conclusion, broad scale patterns of vertebrate richness in the Neotropics is mainly affected by the synergism of the climate and vegetation, followed by the unique contribution of climate. The differences in the relative importance among groups with distinct dispersal abilities indicate that synergistic associations vary according to ecological traits of each vertebrate group. As in most investigations, our study has its own caveats. In defining dispersal ability using vertebrate groups with distinct body sizes, morphologies and physiological constraints, we may have biased our results to some extent. Future investigations could focus on multiple clades within groups relatively homogeneous in their ecological traits [70], and at different spatial scales. Understanding these questions would help to assess the generality of our results, and provide new insights into the interplay between dispersal ability and the environmental drivers of biodiversity patterns.

## Supporting Information

S1 Fig Moran’s Index correlograms for species richness and residuals of the Spatial EigenVector Mapping models used in the variation partitioning analysis. Spatial correlograms for amphibians (A), non-volant mammals (B), bats (C) and birds (D).
(TIF)

S2 Fig Variation in species richness explained by environmental gradients without considering the spatial structure. Primary colors (red, green and blue) denote the proportion of variation explained by the unique fraction of topographic, biotic or climatic sets. Secondary colors (yellow, cyan, magenta) denote the variation commonly explained by two of the three types of environmental set. White color indicates the variation commonly explained by the biotic, climatic and topographic sets. Unexplained variation is omitted for simplicity (see S4 Table for further details on variation partitioning analyses). Each letter on the Venn diagram represents a fraction of the variation partitioning analysis and adds up to the total set of biotic [adfg], climatic [bdeg], and topographic [cefg] factors.
(TIF)

S1 Table Results of principal components analysis using climatic, topographic and biotic sets of variables. (DOCX)

S2 Table Variance Inflation Factor (VIF) and pairwise Pearsons correlations for the first three axes of principal component analysis (PCA) using the climatic, topographic and biotic sets of explanatory variables. (DOCX)

S3 Table Variation partitioning contributions of species richness of Neotropical vertebrates that can be explained by biotic, climatic, topographic, and spatial sets. The identifiable fractions (adjusted R^2^) are designated by lower case letters following the labels displayed in Fig 5.
(DOCX)

S4 Table Variation partitioning contributions of species richness of Neotropical vertebrates that can be explained by biotic, climatic, and topographic sets. The following results were obtained directly by OLS models, without spatial filters. The identifiable fractions (adjusted R^2^) are designated by lower case letters following the labels displayed in S2 Fig.
(DOCX)
