# Supplementary figures and images for: Disentangling the Role of Climate, Topography and Vegetation in Species Richness Gradients
Source: PLoS One. 2016 Mar 25;11(3):e0152468. doi: 10.1371/journal.pone.0152468 (PMC4807822; doi:10.1371/journal.pone.0152468)

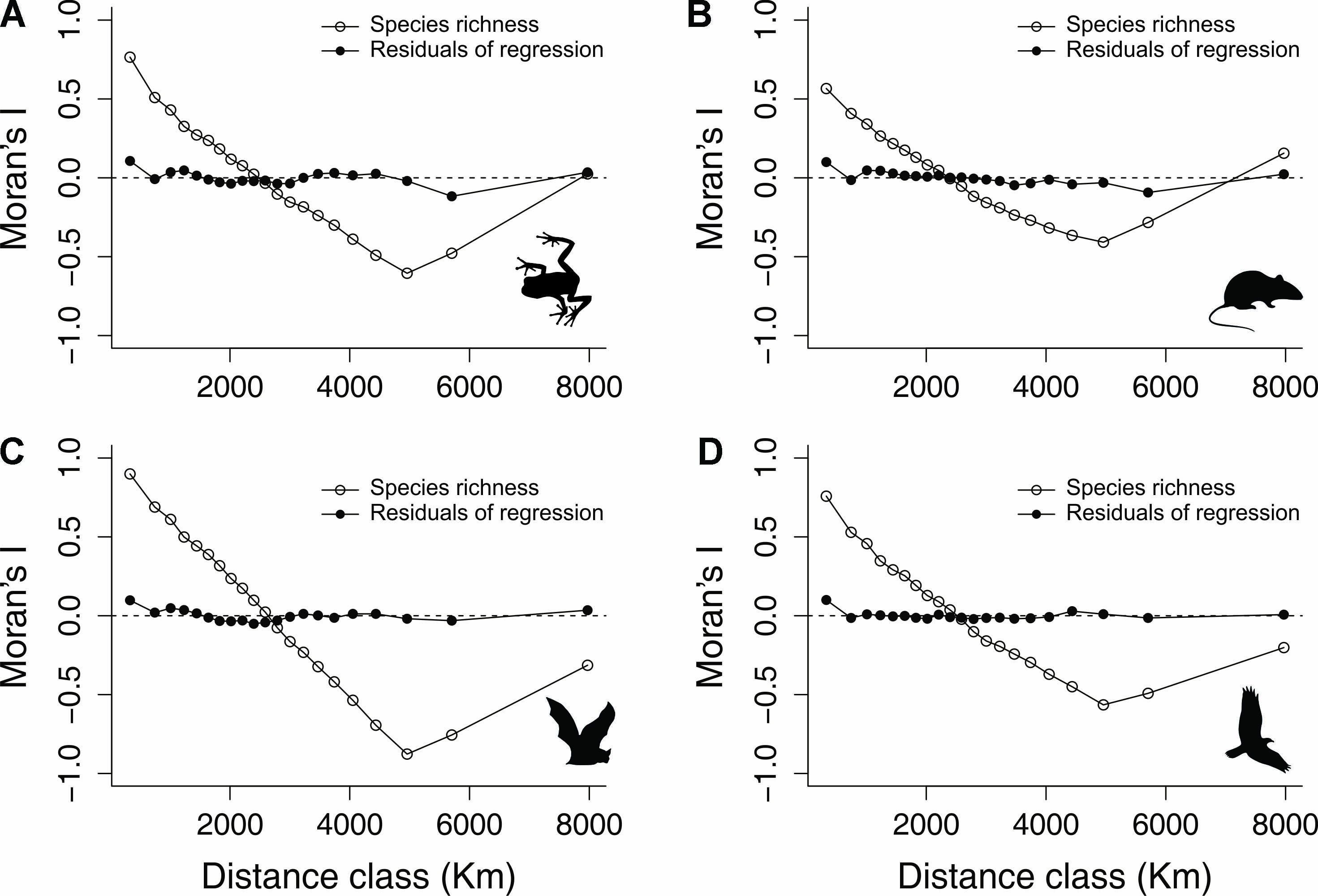

Supplement: S1 Fig — Spatial correlograms for amphibians (A), non-volant mammals (B), bats (C) and birds (D). (TIF) [file pone.0152468.s001.tif]

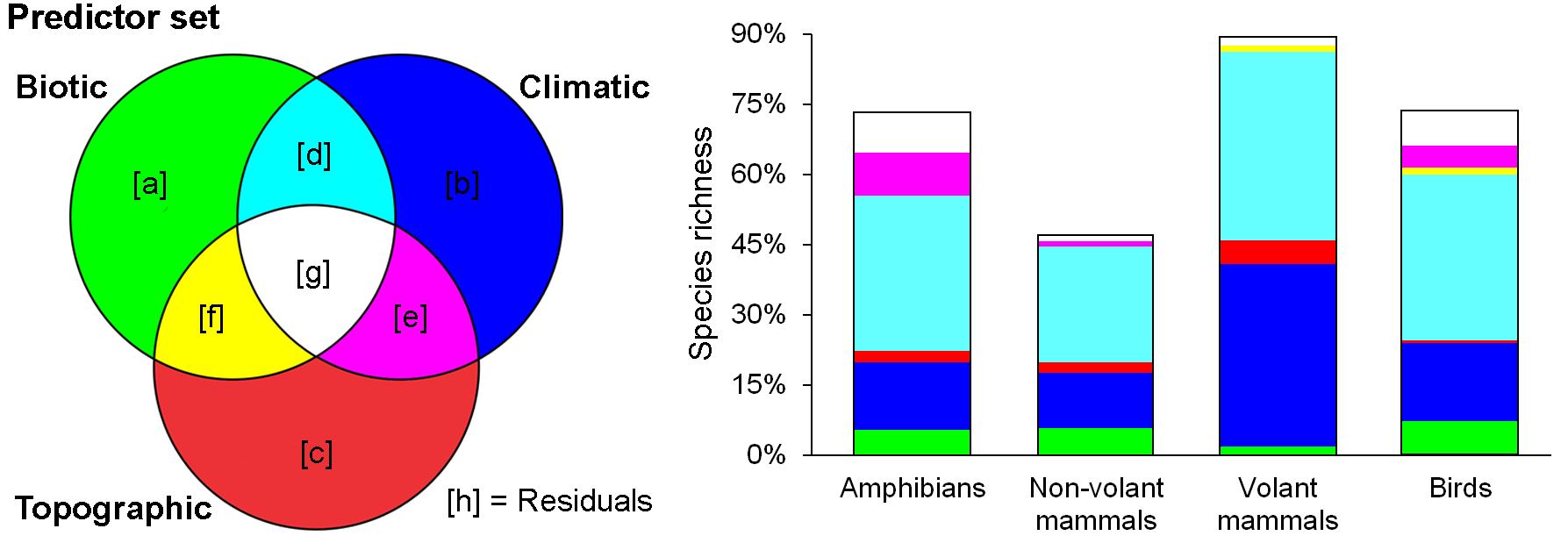

Supplement: S2 Fig — Primary colors (red, green and blue) denote the proportion of variation explained by the unique fraction of topographic, biotic or climatic sets. Secondary colors (yellow, cyan, magenta) denote the variation commonly explained by two of the three types of environmental set. White color indicates the variation commonly explained by the biotic, climatic and topographic sets. Unexplained variation is omitted for simplicity (see S4 Table for further details on variation partitioning analyses). Each letter on the Venn diagram represents a fraction of the variation partitioning analysis and adds up to the total set of biotic [adfg], climatic [bdeg], and topographic [cefg] factors. (TIF) [file pone.0152468.s002.tif]
