# Supplementary material for: Disentangling the Role of Climate, Topography and Vegetation in Species Richness Gradients
Source: PLoS One. 2016 Mar 25;11(3):e0152468. doi: 10.1371/journal.pone.0152468 (PMC4807822; doi:10.1371/journal.pone.0152468)
Supplement: S1 Table — (DOCX) [file pone.0152468.s003.docx]

**Supporting Information to**

Moura, MR; Villalobos, F; Costa, GC; Garcia, PCA. 2016. Disentangling the Role of Climate, Topography and Vegetation in Species Richness Gradients. PLOS One, xxx–xxx.

### S1 Table. Results of principal components analysis using climatic, topographic and biotic sets of variables.

| **Climatic variables** | **PC1** | **PC2** | **PC3** |
| --- | --- | --- | --- |
| AMP | 0.353 | -0.287 | 0.393 |
| PPR | 0.360 | -0.419 | -0.269 |
| TAR | -0.365 | -0.354 | -0.277 |
| APP | 0.375 | 0.289 | -0.304 |
| AMP² | 0.369 | -0.249 | 0.385 |
| TAR² | 0.329 | -0.434 | -0.412 |
| APP² | -0.359 | -0.326 | -0.293 |
| PPR² | 0.314 | 0.420 | -0.447 |
| Proportion of variance (%) | 67.16 | 13.02 | 11.44 |
| Cumulative Proportion (%) | 67.16 | 80.18 | 91.62 |
|  |  |  |  |
| **Topographic variables** | **PC1** | **PC2** | **PC3** |
| ElevM | -0.504 | 0.232 | -0.297 |
| ElevR | -0.515 | -0.132 | 0.398 |
| ElevCV | -0.105 | -0.662 | -0.137 |
| ElevM² | -0.455 | 0.251 | -0.597 |
| ElevR² | -0.504 | -0.073 | 0.516 |
| ElevCV² | -0.088 | -0.650 | -0.335 |
| Proportion of variance (%) | 54.23 | 35.1 | 8.53 |
| Cumulative Proportion (%) | 54.23 | 89.33 | 97.86 |
|  |  |  |  |
| **Biotic variables** | **PC1** | **PC2** | **PC3** |
| FCR | 0.518 | 0.016 | -0.460 |
| FCSD | 0.330 | 0.521 | 0.316 |
| LCD | -0.355 | 0.503 | -0.272 |
| FCR² | 0.512 | -0.011 | -0.498 |
| FCSD² | 0.323 | 0.519 | 0.370 |
| LCD² | -0.360 | 0.454 | -0.480 |
| Proportion of variance (%) | 54.2 | 35.37 | 9.29 |
| Cumulative Proportion (%) | 54.2 | 89.57 | 98.86 |

Abbreviations: AMT = annual mean temperature; APP = annual precipitation; TAR = temperature annual range; PPR = precipitation range; ElevM = mean elevation; ElevR = elevational range; ElevCV = coefficient of variation of elevation; LCD = land cover diversity; FCR = forest canopy height range; FCSD = standard deviation of forest canopy height. The ² denote the quadratic term for the respective variable.
