# Supplementary material for: Disentangling the Role of Climate, Topography and Vegetation in Species Richness Gradients
Source: PLoS One. 2016 Mar 25;11(3):e0152468. doi: 10.1371/journal.pone.0152468 (PMC4807822; doi:10.1371/journal.pone.0152468)
Supplement: S2 Table — (DOCX) [file pone.0152468.s004.docx]

**Supporting Information to**

Moura, MR; Villalobos, F; Costa, GC; Garcia, PCA. 2016. Disentangling the Role of Climate, Topography and Vegetation in Species Richness Gradients. PLOS One, xxx–xxx.

### S2 Table. Variance Inflation Factor (VIF) and pairwise Pearson’s correlations for the first three axes of principal component analysis (PCA) using the climatic, topographic and biotic sets of explanatory variables.

|  | **Clim-PC2** | **Clim-PC3** | **Topo-PC1** | **Topo-PC2** | **Topo-PC3** | **Biotic-PC1** | **Biotic-PC2** | **Biotic-PC3** | **VIF** |
| --- | --- | --- | --- | --- | --- | --- | --- | --- | --- |
| **Clim-PC1** | 0.000 | 0.000 | 0.386 | -0.131 | 0.126 | 0.696 | -0.180 | -0.079 | 2.56 |
| **Clim-PC2** |  | 0.000 | -0.205 | -0.138 | 0.003 | 0.137 | 0.005 | -0.159 | 1.12 |
| **Clim-PC3** |  |  | 0.234 | -0.054 | 0.028 | -0.089 | -0.123 | 0.011 | 1.09 |
| **Topo-PC1** |  |  |  | 0.000 | 0.000 | 0.094 | -0.403 | 0.158 | 1.63 |
| **Topo-PC2** |  |  |  |  | 0.000 | -0.189 | -0.185 | 0.042 | 1.11 |
| **Topo-PC3** |  |  |  |  |  | 0.192 | 0.111 | 0.074 | 1.06 |
| **Biotic-PC1** |  |  |  |  |  |  | 0.000 | 0.000 | 2.26 |
| **Biotic-PC2** |  |  |  |  |  |  |  | 0.000 | 1.29 |
| **Biotic-PC3** |  |  |  |  |  |  |  |  | 1.10 |

Clim-PC, Topo-PC, and Biotic-PC refer to variables based on the first three axes of the PCA using climatic, topographic, and biotic variables, respectively. See S1 Table for individual contribution of each explanatory variable to each principal component.
