# Supplementary material for: Disentangling the Role of Climate, Topography and Vegetation in Species Richness Gradients
Source: PLoS One. 2016 Mar 25;11(3):e0152468. doi: 10.1371/journal.pone.0152468 (PMC4807822; doi:10.1371/journal.pone.0152468)
Supplement: S3 Table — The identifiable fractions (adjusted R2) are designated by lower case letters following the labels displayed in Fig 5. (DOCX) [file pone.0152468.s005.docx]

**Supporting Information to**

Moura, MR; Villalobos, F; Costa, GC; Garcia, PCA. 2016. Disentangling the Role of Climate, Topography and Vegetation in Species Richness Gradients. PLOS One, xxx–xxx.

### S3 Table. Variation partitioning contributions of species richness of Neotropical vertebrates that can be explained by biotic, climatic, topographic and spatial sets. The identifiable fractions (adjusted R²) are designated by lowercase letters following the labels displayed in Fig. 5 (main document).

| **Individual contribution (%)** | **Species richness** | | | |
| --- | --- | --- | --- | --- |
|  | **Amphibians** | **Non-volant mammals** | **Bats** | **Birds** |
| [a] | 2.35 | 3.71 | 1.87 | 4.74 |
| [b] | 15.10 | 17.93 | 27.25 | 14.47 |
| [c] | 0.20 | 2.58 | 2.36 | 0.40 |
| [d] | 16.31 | 30.97 | 6.10 | 12.51 |
| [e] | 29.39 | 18.84 | 32.68 | 32.72 |
| [f] | 5.57 | -0.94 | -0.77 | 5.21 |
| [g] | 0.11 | 0.71 | 0.69 | 1.51 |
| [h] | 3.04 | 1.98 | -0.07 | 2.44 |
| [i] | -0.59 | -5.99 | 11.87 | 2.26 |
| [j] | 2.24 | -0.36 | 2.54 | 0.11 |
| [k] | 3.69 | 5.92 | 7.72 | 2.82 |
| [l] | -0.58 | -0.50 | 0.51 | 3.47 |
| [m] | 3.59 | 1.96 | -2.12 | -0.61 |
| [n] | -1.11 | -0.68 | 0.69 | -0.01 |
| [o] | 9.56 | 1.62 | 1.31 | 4.19 |
| [p] = Residuals | 11.15 | 22.26 | 7.38 | 13.77 |
|  |  |  |  |  |
| Total biotic set [aeghklno] | 46.44 | 31.60 | 45.39 | 51.88 |
| Total climatic set [befiklmo] | 65.72 | 38.84 | 78.43 | 64.54 |
| Total topographic set [cfgjlmno] | 19.58 | 4.39 | 5.21 | 14.28 |
| Total space set [dhijkmno] | 36.72 | 35.41 | 28.04 | 23.71 |

Background colors in the cells of the first column follow the legend color of Fig. 5 (main document).
