# Supplementary material for: Disentangling the Role of Climate, Topography and Vegetation in Species Richness Gradients
Source: PLoS One. 2016 Mar 25;11(3):e0152468. doi: 10.1371/journal.pone.0152468 (PMC4807822; doi:10.1371/journal.pone.0152468)
Supplement: S4 Table — The following results were obtained directly by OLS models, without spatial filters. The identifiable fractions (adjusted R2) are designated by lower case letters following the labels displayed in S2 Fig. (DOCX) [file pone.0152468.s006.docx]

**Supporting Information to**

Moura, MR; Villalobos, F; Costa, GC; Garcia, PCA. 2016. Disentangling the Role of Climate, Topography and Vegetation in Species Richness Gradients. PLOS One, xxx–xxx.

### S4 Table. Variation partitioning contributions of species richness of Neotropical vertebrates that can be explained by biotic, climatic, topographic sets. The following results were obtained directly by OLS models, without spatial filters. The identifiable fractions (adjusted R²) are designated by lowercase letters following the labels displayed in S2 Fig.

| **Individual contribution (%)** | **Species richness** | | | |
| --- | --- | --- | --- | --- |
|  | **Amphibians** | **Non-volant mammals** | **Bats** | **Birds** |
| [a] | 2.35 | 3.71 | 1.87 | 4.74 |
| [b] | 15.10 | 17.93 | 27.25 | 14.47 |
| [c] | 0.20 | 2.58 | 2.36 | 0.40 |
| [d] | 29.39 | 18.84 | 32.68 | 32.72 |
| [e] | 5.57 | -0.94 | -0.77 | 5.21 |
| [f] | 0.11 | 0.71 | 0.69 | 1.51 |
| [g] | -0.58 | -0.50 | 0.51 | 3.47 |
| [h] = Residuals | 11.15 | 22.26 | 7.38 | 13.77 |
|  |  |  |  |  |
| Total biotic set [adfg] | 46.44 | 31.60 | 45.39 | 51.88 |
| Total climatic set [bdeg] | 65.72 | 38.84 | 78.43 | 64.54 |
| Total topographic set [cefg] | 19.58 | 4.39 | 5.21 | 14.28 |

Background colors in the cells of the first column follow the legend color of S2 Fig.
